# The Impact of Smart Pump Technology in the Healthcare System: A Scope Review

**DOI:** 10.7759/cureus.36007

**Published:** 2023-03-10

**Authors:** Fatimah Alamer, Abdullah T Alanazi

**Affiliations:** 1 Health Informatics, King Saud Ibn Abdulaziz University for Health Sciences, Riyadh, SAU; 2 Health Informatics, King Saud Bin Abdulaziz University for Health Sciences, Riyadh, SAU; 3 Research, King Abdullah International Medical Research Center, Riyadh, SAU

**Keywords:** or smart pump safety, smart pump errors, smart infusion pumps, iv smart pump, intravenous smart pumps, smart pump technology, smart pump

## Abstract

Smart infusion pump technology prevents errors caused by parenteral therapy. This paper aims to review the recent literature about smart pump uses, cases and adverse events, and strategies to minimize these disadvantages. Literature was explored from January 2000 to November 2021 using Google Scholar, PubMed, and ScienceDirect. There were assessments of the advantages and adverse effects of using smart pumps and strategies to overcome the adverse effects of smart pumps. The advantage of using smart pumps is that they decrease errors like incorrect rate and dose. Other benefits include a decrease in medication event rates and the ability to connect smart pumps to home health providers. However, compliance rates were negatively influenced by improper smart pumps and the overriding of soft alerts, which can cause alert fatigue and drug library update delays. Recent studies have tried to address the negative issues by improving drug library compliance and decreasing alerts to avoid alert desensitization. The investigations revealed that the smart pumps reduced errors but would only prevent some programming errors. Compliance with utilizing smart pump technology is critical in stopping medication errors. Opportunities for future improvement are broad, including integrating a smart pump infusion with the hospital system, implementing auto programming, and designing smart pump devices to be lighter, smaller, and more portable instead of the heavy, large smart pump used by most hospitals today.

## Introduction and background

Minimizing medical errors is one of the most sought-after goals for modern healthcare organizations. The alarming call of the Institute of Medicine (IOM) in 1999 has urged healthcare organizations to adopt a more conservative approach to dealing with these errors, seeing errors as a system failure rather than an individual's fault. Medication errors could happen in any phase of medication management, namely, prescribing, transcribing, dispensing, and administering the medication. The latest phase needs proper addressing. Technology has been introduced profoundly in the first three phases. Computerized provider order entry (CPOE) was introduced to minimize prescribing errors, and a robotic drug dispenser is another technology that targets dispensing errors.

In addition, a great deal of research has been conducted on the frequency of errors and the ways that can assist in addressing and reducing them in the first three phases. However, medication administration is difficult to track, considering it often happens at the patient’s site. Nevertheless, the medication administration record (MAR) aided with bar-code identification of medication (MAR-BC) has improved patient safety for admitted patients. Medication errors could be multiplied in the cases of specific patient groups (pediatrics, renal insufficiency, and other comorbidity diseases) and critical medications (narrow therapeutic index and parenteral medication). Intravenous medication (IV) administration could seriously injure 60% of patients [1]. Usually, this type of error is a life-threatening one. It should not be tolerated as this route of administration is preserved for critical medication and would reach the patient. Infusion pump technology was introduced to healthcare 45 years ago to monitor the rate and volume of IV medications to enhance safety when administering cardiovascular medication [2]. Providing the right dose is the primary function of the smart pump [3].

In 2018, 89.5% of hospitals in the United States used smart pump technology according to a survey developed by the American Society of Health-System Pharmacists (ASHP). The utilization of smart pump technology varied by hospital bed size with full implementation in hospitals with 600 beds and more [4].

The smart pump contains dose error reduction software (DERS), an essential software proposed to prevent deviations in the infusion programming and warn the users by maintaining drug library standards. A drug library contains drug parameters, dosing limits (minimum and maximum dose), duration rate, and available concentrations. An error in the programmed dose can fire a soft alert that enables the nurse to bypass the alert (soft limit), or a hard alert that does not allow the delivery of medication and the alert cannot be overridden [5]. However, drug libraries are varied among healthcare institutions based on their needs and practice [2]. However, introducing smart infusion pump technologies to hospitals can be overwhelming: smart IV pumps need to be intelligent, require continuous teaching of users, and maintain a precise drug library [6].

Although some evidence supports smart pump technology's effectiveness in eliminating IV drug errors, smart pump systems have different effectiveness rates [7]. With reduced compliance within the drug library, alerts are generated more frequently [8]. This review paper is important as it addresses the status of smart pump technology, determines the advantages and difficulties of the smart pump, identifies strategies to augment the advantages and reduce adverse events, and describes workarounds of smart pump technology.

## Review

Method

Relevant literature was reviewed from January 2000 to November 2019 using different scientific databases (Scopus, PUBMED, Web of Science, and ScienceDirect) using different search strategies, namely the medical subject headings (MeSH) terms (Infusion Pumps/statistics & numerical data, Infusions, Intravenous/methods) and keywords (‘smart pump technology’ OR ‘smart pump’ OR ‘smart-intravenous pumps’ OR ‘IV-smart pump’ OR ‘smart infusion pumps OR Smart pump errors OR smart pump safety). Only studies in English were included. 

Results

Benefits Associated with using Smart Pump Technology

Infusion error prevention: Smart pump infusion technology can potentially reduce/prevent errors like incorrect dose, rate, and pump failure [9]. One study demonstrated an 80% reduction in infusion-related drug errors [9]. A two-year research study by Larsen et al. determined the strength of using smart pumps with a medication library and DERS in the adult intensive care unit (ICU). It has prevented 1136 errors and could avoid at least 300 adverse events in the ICU; 74 errors were among the most severe and had the highest potential to harm the patient [10]. Larsen et al. found a 70% reduction in reporting errors related to basic drug infusions [11].

Impact of smart pumps on adverse drug reactions: A study by Prewitt et al. assessed the patient-controlled analgesia (PCA) adverse events in a pre-post smart IV pump implementation and produced substantially fewer adverse drug reactions [12]. One randomized clinical trial (RCT) by Rothschild et al. assessed the impact of smart infusion technology on the intention to treat adverse events. The reduction was 0.18 for every 100 pumps per day. Nevertheless, the incidence of treating adverse drug events was not statistically significantly reduced after implementing smart infusion technology [13].

Smart pump technology with home health provision: Unlike the smart pump infusion used in hospitals, home healthcare providers utilize traditional infusion pumps, which do not have a drug library or drug-safe software. Therefore, the impact of the smart pump infusion is controversial. Nevertheless, upon training home health care providers on ambulatory infusion pumps and introducing a particular drug library by clinicians, the impact was feasible for home infusion [14]. In doing so, patients were satisfied and could respond to alerts without contacting home healthcare providers [14].

Difficulties of the Smart Pump Infusion

Smart pump infusion errors: The smart pump technology cannot stop all the infusion regarding drug errors and could make new error types. Smart pump technology has an insufficient effect without DERS utilization. Before and after the study, there was almost an 80% decrease in errors when utilizing the DERS, but the difference was not significant without using it [9]. The most common-reported variety of IV administrating errors were: medication not dispensed (23%) due to open tubing failures mainly when administering a secondary infusion, wrong pump rate (almost 20%), an incorrect drug (17%), or incorrect dose (14%) [15]. 

In one observational research of IV preparation and administrating drugs in an ICU of a teaching hospital, the majority of errors were that bolus doses were administered faster than the actual recommended rate. In another study of IV drug administration, bolus administration was linked with a 31% expanded error risk [16]. The basic infusion profile delivered more than half of smart pump infusions. However, basic infusion lets clinicians bypass medication limits by skipping the name of the medication. No alerts can be made by continuous infusions if bypassing drug limits. One of the risk factors for alerts with infusions is the nursing shift change. The odds of infusion rates with alerts were 1.3 times greater than infusions in the day shift [17]. Most of the second medication administration is created to permit the primary infusion to restart if the secondary infusion is completed [18].

Drug library updates delays and compliance: Wireless drug library update delay for a smart pump is common, which may harm patients if a pump is designed with the wrong limit frame while administrating medication. Retrospective research was done to measure medication library update delays over two years in 49 hospitals using 12 health systems. Eleven health systems had substantial drug library updates from 22 to 192 days [19]. Health systems must be attentive to the significance of smart-pump update delays to decrease harmful events. The bypassing library may differ based on the medication involved. In the RCT by Prewitt et al., nurses neglected the medication library in about 70% of propofol infusions and about 60% of insulin infusions, with a 25% bypass rate [12].

Override of soft alerts: Most alerts are ignored at the point of care, which adds to the problem of alert fatigue problems. Although high alert drugs (HAD) could illustrate a significant harmful risk, one study demonstrated 75.8% of the alerts are bypassed in 15 hospital systems. Bypassed alerts did not reduce IV drug error since they did not alter the intended drug administration [18].

Strategies to Overcome the Negative Impact of Smart Pump Technology

Enhancing drug library compliance: It is critical to ensure safety, thus, non-compliance with the medication library can be contrary to outcomes, effects, and quality of interest. Drug library compliance was measured by reporting real-time applications. Thus, pharmacists and nurses were directly aware of any programmed infusions not in the drug library, which allowed them to be useful succeed. Real-time units resulted in 100% compliance by utilizing the medication library in all six ICUs. However, information from the other departments bypassed the medication library 34% of the time. The causes of ignoring the drug library were determined. Some infusion borders were recognized as an extreme restriction, so a medication library was modified to decrease alert fatigue and drug non-compliance. In addition, training for medication library use during yearly skills authorization for documentation of a continual increment in house-wide medication library compliance and a total reduction in alert tendency is essential [8].

Alert reduction: Alerts from the smart pump deliver a caution if a nurse tries to set limits outside the range of hospital dosing standards. Alert fatigue is contributed to a high incidence of alerts inducing clinicians to ignore warnings. In one study, alert frequency while starting the use of a new smart pump technology was about 4%; almost more than half of the medication in the library had alerts, but the rate declined in the next three months to 1.16% of alerts. The first three months of using smart pumps were used to identify increased alert rates and apply recommendations to decrease alerts and enhance medication library compliance. For instance, in the case of soft limit overrides, maximizing the soft limit of heparin units from 1500 to 1800 per hour reduces about 55% of correlated overrides. Two override issues have also been identified. The first issue is the selection error i.e., selecting a wrong medication entry when various entries were available for the same infusion. The second issue is the bolus dosing, which increased the infusion rate instead of utilizing the bolus advantage to build safe borders. This is a clear example of optimizing the patient’s safety in the drug library [8].

Error reduction: Responses to alerts that are managed and corrected may simplify a second checking for nurses, or they could perform correction of mistakes in programming. The decreased number of corrections means suitable dosing by the nurses. Top smart pump infusions for correcting the doses include: heparin, phenylephrine, and potassium chloride piggyback. There were 13 dose corrections of heparin because of both over- and under-dosing. The drug could cause harm to the patient and financial setbacks to the hospital if there were no dose corrections. The 13 correction doses saved $113,750 [8]. 

Discussion

The smart pump is a technology that manages most negative results associated with utilizing IV drugs, which can be harmful. During the administration phase, errors can also happen when using smart pumps, like identification of patient errors, documentation errors, or labeling errors on IV drugs [18,20]. The ordering and the preparation of drug phases are essential components of utilizing smart pumps. A decreased medication library compliance rate can obstruct the efficacy of smart pumps. A high number of alarms, which happened once in three uses of smart pumps, means there are more than 106 hours of alarms per month for one healthcare institution [17]. Bypassing alerts from the drug library is another issue that could minimize the safety advantages of the smart IV pump. Some research has highlighted causes for the overriding medication of libraries, such as a false low-risk perception, a failure to change the medication library when an alert is unreliable, and the necessity for extensive work to utilize the smart pump technology, work tension, and emergencies [20]. However, new studies have tried to address solutions for problems that can occur with smart pumps, which need careful and systematic implementation. For example, an alert reduction is vital to avoid alert fatigue that can cause clinicians to bypass alerts or act incorrectly by adjusting alarm limits beyond the safe range to decrease alarm numbers. 

Giuliano has confirmed the need for innovation for the smart pump technology that includes: auto-programming, which means that orders of a drug are sent immediately to the infusion pumps from the pharmacy information system and then approved by a clinician before starting the infusion; auto-documentation of the infusion smart pump program in electronic information systems [15]. System integration in information technology for ordering, standardization control to decrease misunderstanding, fluctuation of function interpretation of products from various sources, and enhancements to screen visibility and size are some of the insufficient features of the smart pump that require improvements to help clinicians view information to better infusion transmission. Also, smart pump devices should be lighter, minimal, and transportable.

## Conclusions

The current literature review shows smart pump technology boosts safe administration and prevention of infusion errors. The evidence shows an improvement in smart pump technology. Smart pumps can decline error rates of programming but some errors happen after introducing smart pump technology to hospitals, like medication drug administration and incorrect patient information. Still, an absence of integration with hospital systems may decrease the support of smart pumps as drug library compliance is critical for a successful smart pump infusion. Each hospital has to enhance the rate of compliance using the pump technology and the medication library to work as desired by the proposed strategies, progression, upgrading, and development of medication libraries. Therefore, keeping a current medication library is recommended to maximize reducing errors, avoid drug update delays, and avoid alarm fatigue that could occur from several factors such as alarm desensitization. Smart pump technology grows rapidly as new merits are introduced, like adjusting a drug library to be suitable for home use and a program for alert reduction. However, there is still a necessity for innovation in smart IV pumps technology, such as auto-programming and auto-documentation, and decreasing the size of a smart device to make it easily portable. 
